# Supplementary material for: The In Vitro and In Vivo Synergistic Antimicrobial Activity Assessment of Vacuum Microwave Assisted Aqueous Extracts from Pomegranate and Avocado Fruit Peels and Avocado Seeds Based on a Mixtures Design Model
Source: Plants (Basel). 2021 Aug 24;10(9):1757. doi: 10.3390/plants10091757 (PMC8471749; doi:10.3390/plants10091757)
Supplement: Supplementary file 1 [file plants-10-01757-s001.zip › plants-1344728-supplementary.pdf]

Table S1. Nutrition Declaration per 100g of the foods used in the food models.

	**Minced beef meat**	**Cheese salad dressing**	**Greek Yogurt**
Energy	1,387kJ /332kcal	842kJ /201kcal	536kJ /129kcal
Total Fat	30g	16.1g	10g
Saturated Fat	11.9g	4.9g	7.5g
Carbohydrates	0g	4g	3.5g
Sugars	0g	2.5g	3.5g
Proteins	14g	9.1g	5.5g
Salt	67mg	2g	110mg
